# Tolerance of two anhydrobiotic tardigrades *Echiniscus testudo* and *Milnesium inceptum* to hypomagnetic conditions

**DOI:** 10.7717/peerj.10630

**Published:** 2021-02-02

**Authors:** Weronika Erdmann, Bogdan Idzikowski, Wojciech Kowalski, Jakub Z. Kosicki, Łukasz Kaczmarek

**Affiliations:** 1 Department of Animal Taxonomy and Ecology/Faculty of Biology, Adam Mickiewicz University of Poznan, Poznań, Poland; 2 Institute of Molecular Physics, Polish Academy of Sciences, Poznań, Poland; 3 Department of Avian Biology and Ecology/Faculty of Biology, Adam Mickiewicz University of Poznan, Poznań, Poland

**Keywords:** Extremophiles, Geomagnetic field, Geomagnetobiology, Invertebrates, Astrobiology, Dehydratation, Magnetic field

## Abstract

The open space is a hostile environment for all lifeforms not only due to vacuum, high radiation, low atmospheric pressure, and extremely low temperature, but also the absence of the geomagnetic field. The geomagnetic field protects Earth mainly from corpuscular radiation, that is, solar wind and cosmic radiation, but above all it influences organisms, including their cells, tissues and organs. Moreover, numerous studies conducted on plants and animals confirmed that hypomagnetic conditions (the term referring to all situations when the magnetic field is weaker than the typical geomagnetic field) have significant influence on the metabolism of living organisms. Although many studies dealt with a variety of aspects related mainly to the influence of hypomagnetic conditions on human health. Very few studies have considered the influence of hypomagnetic conditions on extremophiles. Astrobiologists have long been testing different extremofiles to find out if any multicellular organisms are able to survive in extreme conditions of open space. Among all multicellular extremophiles fit for such research, water bears (Tardigrada) are the most interesting. Not only are they one of the most resistant organisms on Earth, but results obtained from studies on these invertebrates can be extrapolated or applied to vertebrates (including humans). Despite this, studies on the influence of hypomagnetic conditions on tardigrades are rare, so far. In the present study, to test the influence of hypomagnetic conditions on the process of anhydrobiosis while entering and returning from anhydrobiosis, we used two terrestrial anhydrobiotic species that are *Echiniscus testudo* and *Milnesium inceptum*. To exclude the ambient magnetic field, experiments were carried out in a special magnetic field shielding chamber. In total, three experiments were conducted: (a) tardigrades in anhydrobiosis, (b) tardigrades entering anhydrobiosis and (c) tardigrades returning to active life. The obtained results clearly showed that even partial isolation from the geomagnetic field, that is, hypomagnetic conditions, has negative influence on anhydrobiotic abilities of both tested tardigrade species. In both species we observed lower survivability rate while entering anhydrobiosis, in anhydrobiotic state and returning to the active state. What is more, we observed a higher mortality rate in *Ech. testudo* than *Mil. inceptum* which suggest that different species response to the hypomagnetic conditions in different way. In conclusion, while current knowledge on the influence of hypomagnetic conditions on mortality of invertebrates is very limited, our results suggest that the presence of the magnetic field is a very important factor which should be considered in further research focused on potential survival of Earth organisms in outer space, spacecrafts or different planets and moons.

## Introduction

Outer space is a hostile environment for all lifeforms not only due to vacuum, high radiation, low atmospheric pressure, and extremely low temperature (Chuss, 2008), but also the absence of the geomagnetic field (Hargreaves, 1995; Sibeck & Lin, 2014). Astrobiologists have long been testing different organisms to find out if any multicellular organisms are able to survive in such extreme conditions (Langbein, 1986; Gmünder et al., 1989; Morrow et al., 1994). These studies are important from two points of view: first, it is important to identify organisms that are able to survive in unfavourable conditions, for example, on different planets or moons; second, we need to identify organisms which can potentially contaminate space probes sent to other celestial bodies and subsequently falsify research on search for extra-terrestrial life (Caplin, 2019; Vidaurri, 2019). Discussion focusing on interplanetary contamination, that is, the risk of transferring different lifeforms or organic material to an explored celestial body, are not new as it started in the late 1950s (National Research Council, 2006). As a result, broad recommendations for planetary protection were included in Article IX of the United Nations Outer Space Treaty from 1967 (National Research Council, 2006). Moreover, the Committee on Space Research (COSPAR) prepared strict recommendations for the sterilisation of spacecraft, landers and rovers (Committee on Space Research (COSPAR), 2002; National Research Council, 2006). Even if such distant missions with living organisms on-board, also extremophiles, have been rare, but we cannot exclude that they will be planned in the future (Erdmann & Kaczmarek, 2017). Still, despite many doubts and potential problems, we cannot abandon missions involving living organisms, because they are too important in the context of future space exploration. Studies conducted on cells, tissues and entire living organisms (active or cryptobiotic) are crucial for the future of manned space missions to Mars and other locations in the Solar System. For such missions, we need to know the limits within which living organisms can survive. What is more, such knowledge is necessary to define the probability of finding extraterrestrial life.

Among all multicellular extremophiles fit for such research, tardigrades are the most interesting. Not only are they one of the most resistant organisms on Earth (Copley, 1999), but results obtained from studies on these invertebrates can be extrapolated or applied to vertebrates, including humans (Hashimoto et al., 2016).

Tardigrada or water bears are microinvertebrates (mean size ca. 500 μm) which inhabit almost all terrestrial and aquatic ecosystems throughout the world (Nelson, Guidetti & Rebecchi, 2015). On Earth, in laboratory conditions tardigrades are able to survive many physical and chemical stressors, such as low and high temperatures, high doses of radiation, drying, low and high atmospheric pressure, low gravity, high concentration of heavy metals, toxins like CO_2_ or ethidium bromide (see reviews, for example, Ramlov & Westh, 2001; Altiero et al., 2011; Guidetti et al., 2012; Erdmann & Kaczmarek, 2017; Kaczmarek et al., 2019). However, in the context of astrobiology, the most important question is if tardigrades are able to survive in all these stress conditions also in extraterrestrial environments. Although it was shown that some tardigrades are able to survive even the exposure to the space vacuum (Jönsson et al., 2008; Rebecchi et al., 2009a, 2009b; Persson et al., 2011; Rebecchi et al., 2011a, 2011b; Vukich et al., 2012), the experiments were conducted within the protection of Earth’s magnetic field, i.e. on the low Earth orbit (ESA, official materials for FOTON-M3 Mission, http://esamultimedia.esa.int/docs/foton/FOTON-M3_brochure.pdf).

The magnetic field is absent or much weaker in the outer space and on some planets and moons, such as Venus, Mars, the Moon, which do not generate their own inner magnetic field. Although this fact has been known for a long time, the absence of the geomagnetic field has been ignored in all astrobiological experiments conducted on tardigrades and other extremophiles. The term ‘hypomagnetic conditions’ refers to all situations when the magnetic field is weaker than the typical geomagnetic field (GMF), including its complete absence, which is one of characteristics of the outer space outside Earth’s magnetosphere. Due to the solar wind, Earth’s magnetosphere is asymmetrical and it reaches ca. 65,000 km (10 Earth’s Radius) from Earth’s surface on the side faces the Sun (Hargreaves, 1995); whereas on Earth’s nightside, the magnetic field extends into magnetotail, measuring from 6.3 × 10^5^ km (100 Earth’s Radius) in the near-magnetotail to ca.12.6 × 10^5^ km in the distant magnetotail (Sibeck & Lin, 2014). The GMF protects Earth mainly from corpuscular radiation, that is, solar wind and cosmic radiation, but above all it influences organisms, including their cells, tissues and organs (Dubrov, 1978; Conley, 1970; Tombarkiewicz, 2008; Janicki, 2008; Baek et al., 2019). The GMF is also a source of information for many organisms (mainly animals) whose abilities include magneto-detection and magneto-navigation (Wiltschko & Wiltschko, 2005; Brothers & Lohmann, 2015; Yosef et al., 2020). That is why it comes as no surprise that hypomagnetic conditions have a strong negative impact on Earth’s organisms (Dubrov, 1978; Conley, 1970; Janicki, 2008; Tombarkiewicz, 2008).

Studies on organisms’ tolerance to long-term isolation from Earth’s magnetic field are particularly important for astrobiology and space medicine (Dubrov, 1978; Grigoriev & Potapov, 2014). Their heyday was in the 1970s when the influence of hypomagnetic conditions on living organisms was analysed, including space flight conditions (Conley, 1970; Dubrov, 1978). In those and later studies researchers dealt with a variety of aspects related mainly to the influence of hypomagnetic conditions on human health (Dubrov, 1978; Binhi & Sarimov, 2013; Mo, Liu & He, 2014; Mo et al., 2014; Gurfinkel et al., 2016) or on animal’s metabolism (mostly mice and rats) (Conley, 1970; Tombarkiewicz, 2008). All of these studies clearly demonstrated that a long-term exposure to hypomagnetic conditions had a significant influence on the tested organisms, reducing their fitness and activity (Conley, 1970; Tombarkiewicz, 2008; Ding et al., 2018), hypomagnetic conditions also affected the functioning of the brain (Zaporozhan et al., 2002; Mo, Liu & He, 2014; Wei-Chuan et al., 2015 ), that is, in humans by deteriorating cognitive abilities (Sarimov, Binhi & Milyaev, 2008; Binhi & Sarimov, 2009; Binhi, 2012). Moreover, long-term exposition to hypomagnetic conditions leads to circadian rhythm disorder (Gurfinkel et al., 2016), decreased metabolism, gastrointestinal diseases, and a decreased number of leukocytes (Janicki, 2008). Embryonic development was negatively affected because of inhibited early embryogenesis and reproduction capacity (Wang et al., 2003; Zhang et al., 2004; Osipenko et al., 2008; Xiao et al., 2009; Fesenko et al., 2010). Although studies on the influence of hypomagnetic conditions on humans and other vertebrates have been quite common, similar attention to invertebrates is still scarce (Dubrov, 1978).

So far, studies on the influence of hypomagnetic conditions on tardigrades are limited to only one paper published by Erdmann et al. (2017). A research on eutardigrade *Hypsibius dujardini* (Doyère, 1840) (recently redescribed under the name *Hys. exemplaris* Gąsiorek et al., 2018 and differentiated from *Hys. dujardini* sensu stricto) shows that in hypomagnetic conditions its mortality is much higher while entering anhydrobiosis or returning to the active state (Erdmann et al., 2017). On the basis of the obtained results, a hypothesis was formed that certain metabolic processes associated with anhydrobiosis could be disturbed or impaired by hypomagnetic conditions, for example some changes appeared in the expression of stress proteins, but the results were not conclusive (Erdmann et al., 2018). Besides, *Hys. exemplaris* used in those studies was not the best candidate for testing its anhydrobiotic abilities, because as an aquatic species its ability to survive in anhydrobiotic conditions is low (Wright, 1989; Kondo, Kubo & Kunieda, 2015; Boothby et al., 2017; Boothby, 2018).

In the present study, to test the influence of hypomagnetic conditions on the process of anhydrobiosis while entering and returning from anhydrobiosis, we used terrestrial tardigrade species, such as Heterotardigrada *Echiniscus testudo* (Doyère, 1840) and Eutardigrada *Milnesium inceptum* Morek et al. (2019), whose anhydrobiotic abilities are very high (Wright, 1989; Jönsson, Borsari & Rebecchi, 2001; Rebecchi et al., 2006; Jørgensen, Møbjerg & Kristensen, 2007; Jørgensen et al., 2013; Roszkowska et al., 2018). We studied meticulously how hypomagnetic conditions affected survivability of both terrestrial species at their different physiological stage (active vs in anhydrobiosis) and then compared the results with results obtained for aquatic *Hys. exemplaris*.

## Materials and Methods

### Samples processing

Two tardigrade species were collected in a xerothermic habitat that is, mosses on concrete wall in the city centre of Poznań in Poland (Heliodor Święcicki Clinical Hospital of Poznań University of Medical Sciences at Przybyszewskiego street, 52°24′15″N, 16°53′18″E; 87 m asl). Tardigrades were obtained from samples according to a standard procedure (Stec et al., 2015). Moss samples were placed in plastic beakers containing 250 ml of spring water (Żywiec Zdrój) mixed with distilled water (in ratio 1:3), called the medium. After 24 h, the mosses were strongly shaken with tweezers and all plant particles were removed. The water containing tardigrades was poured into a 250 ml plastic cylinder. After 30 min the upper portion of water (ca. 200 ml) was decanted, and the remaining 50 ml was poured into Petri dishes, while living tardigrades were extracted under a stereomicroscope (Olympus SZ51) and transferred to small Petri dishes with clean water.

### Model species

*Echiniscus testudo* is a widely distributed parthenogenetic, herbivorous and terrestrial heterotardigrade species (McInnes, 1994; Jørgensen, Møbjerg & Kristensen, 2007; Jørgensen et al., 2013; Kaczmarek, Michalczyk & McInnes, 2015, 2016; McInnes, Michalczyk & Kaczmarek, 2017; Gąsiorek, Vončina & Michalczyk, 2019), while *Mil. inceptum* is a dioecious (but it can also reproduce parthenogenetically), predatory and terrestrial Eutardigrade species known from few distant localities (Roszkowska et al., 2018; Morek et al., 2019). Both species inhabit very dry xerothermic mosses or/and lichens. Due to the fact that the methodology for culturing Heterotardigrada is not known, specimens of *Ech. testudo* were extracted directly from the samples. They were kept two additional hours in a clean water and then these specimens were segregated and only fully active adult specimens of medium body length were selected for experiments. Fully active specimens of *Mil. inceptum* were extracted directly from the sample and a laboratory culture was established. Parthenogenetic *Milnesium* females were cultured in the medium on scratched Petri dishes where rotifers (*Lecane inermis* Bryce, 1892) and nematodes (*Caenorhabditis elegans* Maupas, 1900) were added ad libitum as a food source. Fully active, adult and not moulting females of medium body size were selected for experiments. Before experiments, the chosen tardigrades were kept two additional hours in clean water.

### Anti-magnetic chamber and control box

**Figure 1:**
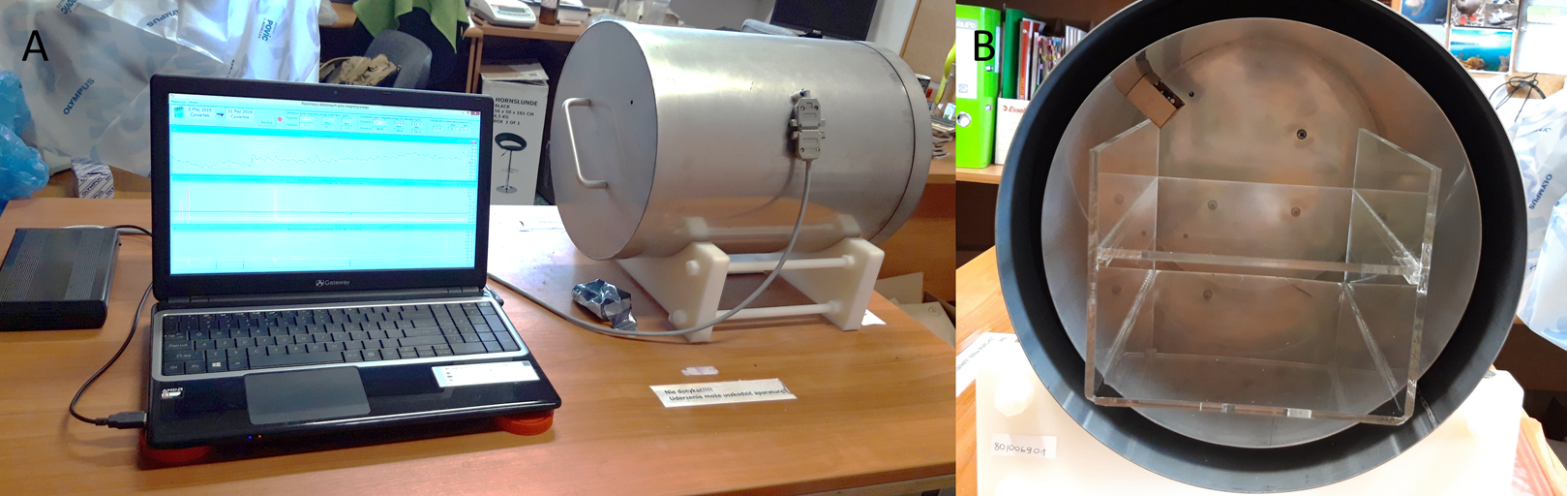
Chamber Isolated from the Magnetic Field (CIMF). (A) CIMF chamber during work, connected with a laptop, (B) inside view of the CIMF chamber, with shelves for test objects. The internal size of CIMF is 26.2 cm in diameter and 35 cm in length.

To reduce the ambient magnetic field, all experiments were carried out inside a special magnetic field shielding chamber (Chamber Isolated from Magnetic Field—CIMF) constructed and used by our team in our previous studies on *Hys. exemplaris* (Erdmann et al., 2017). The CIMF is a double cylinder closed at both ends with a double-layered lid, with internal size of 26.2 cm of diameter and 35 cm of length (Fig. 1B), made of a mu-metal (μ-metal) tinware (for more details see Erdmann et al., 2017). Like some other iron or nickel-based amorphous and/or nanocrystalline alloys with Zr, Nb or Mo additions (Kopcewicz et al., 1999; Miglierini et al., 2002; Idzikowski et al., 2004), mu-metal is easily saturated in static or slowly varying magnetic fields, which is appropriate for use as a shielding material.

The geomagnetic field inside and outside the chamber and the temperature inside the CIMF were measured and later transmitted for analyses through a USB interface to the computer (for more details see Erdmann et al., 2017) (Fig. 1A).

Following the same protocol as in our previous work (Erdmann et al., 2017) to provide conditions similar to those inside the CIMF, but without hypomagnetic condition, for control groups, the Control Box was used. As the Control Box is a plastic container of the size of 18.5 × 12 × 33 cm, uninsulated from the magnetic field.

### Experiments methodology

To test the influence of hypomagnetic conditions on the process of anhydrobiosis of *Ech. testudo* and *Mil. inceptum*, three experiments were conducted: (I) with specimens in anhydrobiosis, (II) with specimens entering anhydrobiosis, and (III) with specimens returning to active life.

In every experiment 200 specimens (100 in the control group and 100 in the test group) of both species were used. In each experiment, 10 replicates were performed, with 10 specimens in each Petri dish (both in control and test groups).

All selected animals were placed in plastic Petri dishes (ø = 40 mm) on filter paper (grammage 85–87 gsm, Chemland Company) moistened with 450 microliters of the medium (spring water “Żywiec Zdrój” mixed with distilled water in ratio 1:3). Dehydration of all specimens took 3 days in the average temperature of 23.7°C (Data S1). As we explained in our previous work “Can the tardigrade *Hypsibius dujardini* survive in the absence of the geomagnetic field?” additional monitoring equipment (i.e., electronic hygrometers) cannot be used as it could increase the level of the magnetic field in the chamber. Therefore, humidity level inside CIMF and Control Box was not controlled nor modified (Erdmann et al., 2017). Air humidity in both CIMF and Control Box was approximately the same as in the laboratory and range between 40% and 60%.

Because we wanted our newest results to be comparable to the results of our older studies on *Hys. exemplaris* (Erdmann et al., 2017) we adapted as much as possible procedures used in those earlier studies. All experiments were conducted for 21 days in the same laboratory room. Test groups were kept either in the CIMF or—when necessary (when tested specimens should be kept in normal magnetic conditions)—in a box that was identical to the Control Box. Control groups were kept in the Control Box. However, a different procedure for rehydration was used, because the newly tested species do not need as long rehydration time as, previously used *Hys. exemplaris*. Rehydration was conducted, for 1 h, in the 450 microliters medium which was added to dried Petri dishes. After this time, specimens were observed continuously for the following 2 h, with three hours in total time spent, by specimens, in the medium Later, specimens were divided into dead (without signs of movements of the body, legs or internal structures) or alive (with at least some signs of movements).

To measure the rate of tardigrade mortality (expressed as the percentage of dead individuals) in all experiments both dead and specimens were counted and typed into databases, first one for *Ech. testudo* (Data S2), and second for *Mil. inceptum* (Data S3).

*Experiment I*. In this experiment, we tested the influence of hypomagnetic conditions on anhydrobiotic individuals of *Ech. testudo* and *Mil. inceptum*. The experiment was conducted as previously described in Erdmann et al. (2017). Specifically, specimens from test groups were dehydrated on Petri dishes and later placed in the CIMF for 21 days. After this time, they were removed from the chamber and rehydrated on the same Petri dishes. Concurrently, the dehydrated specimens from the control group spent 21 days in the Control Box. After this time, all specimens from both the CIMF and the Control Box were rehydrated and living and dead specimens from both groups were counted.

*Experiment II*. In this experiment we, tested the influence of hypomagnetic conditions on the process of entering anhydrobiosis. Following the procedure form our previous work (Erdmann et al., 2017) specimens in the tested groups were dehydrated inside the CIMF, and then Petri dishes with tardigrades were removed from the chamber. The specimens spent 21 days in this state under the influence of the geomagnetic field in the Control Box in the same conditions as the control group. The specimens from the control group were dehydrated in the geomagnetic field, and then spent 21 days in the Control Box. After this period, all specimens were rehydrated in normal geomagnetic field conditions. Living and dead specimens from both groups were counted.

*Experiment III*. In this experiment, we tested the influence of hypomagnetic conditions on tardigrades in the process of returning from anhydrobiotic to the active state. The experiment was conducted as previously described in Erdmann et al. (2017). Specifically, specimens of both tested species were dehydrated outside the CIMF and spent 21 days in the anhydrobiotic state under the influence of the geomagnetic field. Subsequently, Petri dishes with tested specimens were transferred to the CIMF, and specimens were rehydrated in the medium in hypomagnetic conditions Specimens from the control group spent 21 days after dehydration in the Control Box, and then were rehydrated in the same conditions. After rehydration, all specimens were rehydrated and living and dead specimens from both groups were counted.

### Statistical analysis

In all three experiments, tardigrade mortality in each Petri dish was calculated (Datas S2 and S3). To test the differences in mortality between both groups, that is, experimental vs control, we used the *t-test* with the Cochran-Cox adjustment, whereas differences between particular experiments were tested with the one-way ANOVA (Zar, 1999) with the Tukey test as a post-hoc. In the latest case, the control group is expressed as the minimum mortality on each dishes for each control groups from three experiments (mean ± SE of *Ech. testudo* = 9.0 ± 2.3, mean ± SE of *Mil. inceptum* = 7.0 ± 2.1). All calculations were performed with the R 3.2.3.

To check whether mortality of the tested tardigrade species did not fluctuate even in normal magnetic field conditions, the one-way ANOVA test was carried out for test groups of both species alike. No significant differences were found in mortality of control groups in all three experiments for either *Ech. testudo* (one-way ANOVA: *F*_2,27_ = 0.97, *p* = 0.39) or *Mil. inceptum* (*F*_2,27_ = 1.25, *p* = 0.21).

## Results

### Experiment I: testing the influence of hypomagnetic conditions on anhydrobiotic individuals

**Figure 2:**
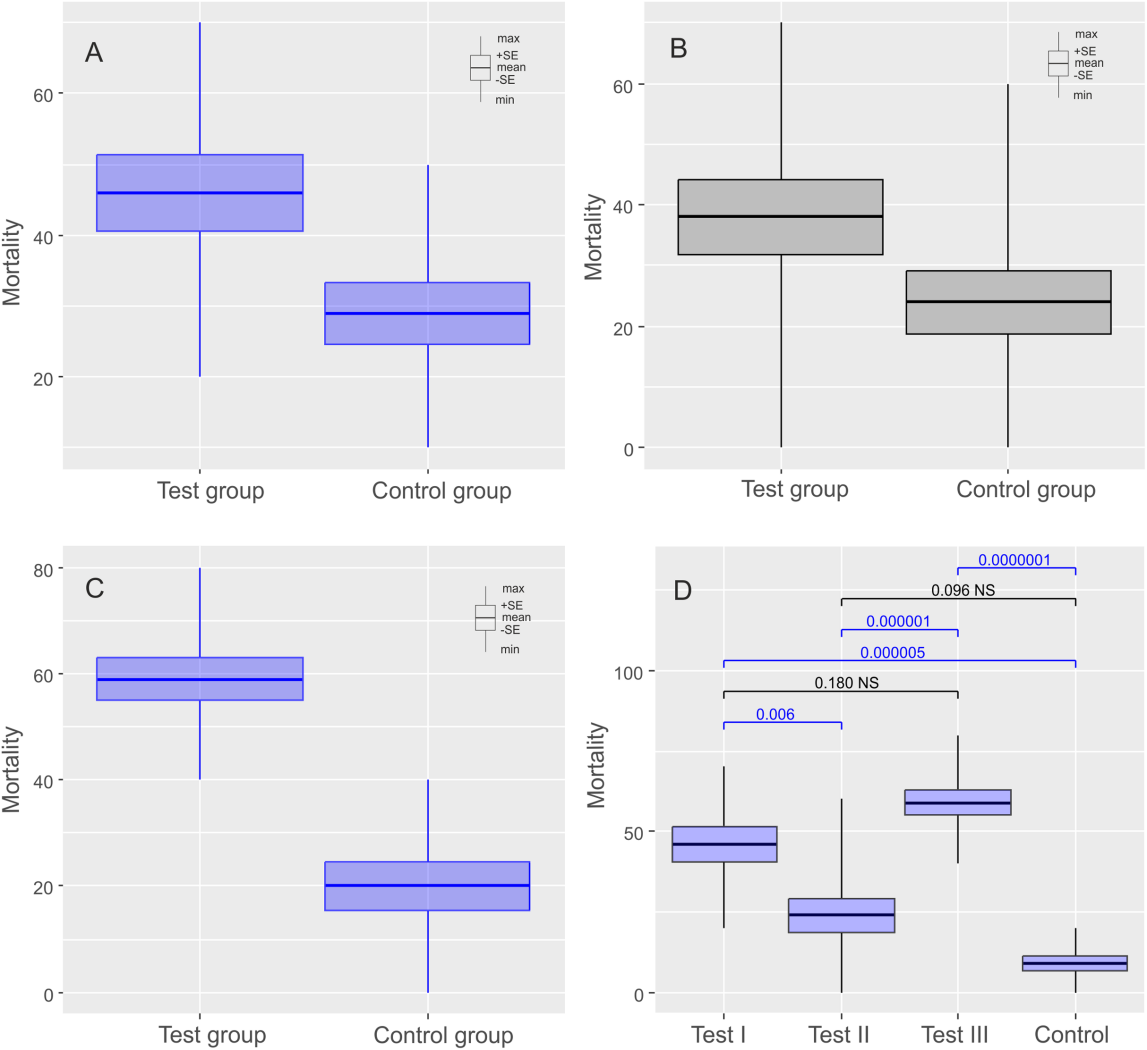
Results of experiments on *Echiniscus testudo* Doyère, 1840 shown as comparisons of mortalities in test and control groups. The diagrams show (A) comparisons of mortalities of anhydrobiotic specimens, (B) comparisons of mortalities of the specimens during entering into cryptobiosis, (C) comparisons of mortalities of the specimens returning to an active state, and (D) the comparison of experiments. Every diagram shows the difference in mortality (expressed as the percentage of dead individuals) between the Test Group and the Control Group. Blue indicates statistically significant differences, while gray indicates statistically insignificant differences. Max, maximal value; Min, minimal value; Mean, mean value and SE, stands for standard error.

**Figure 3:**
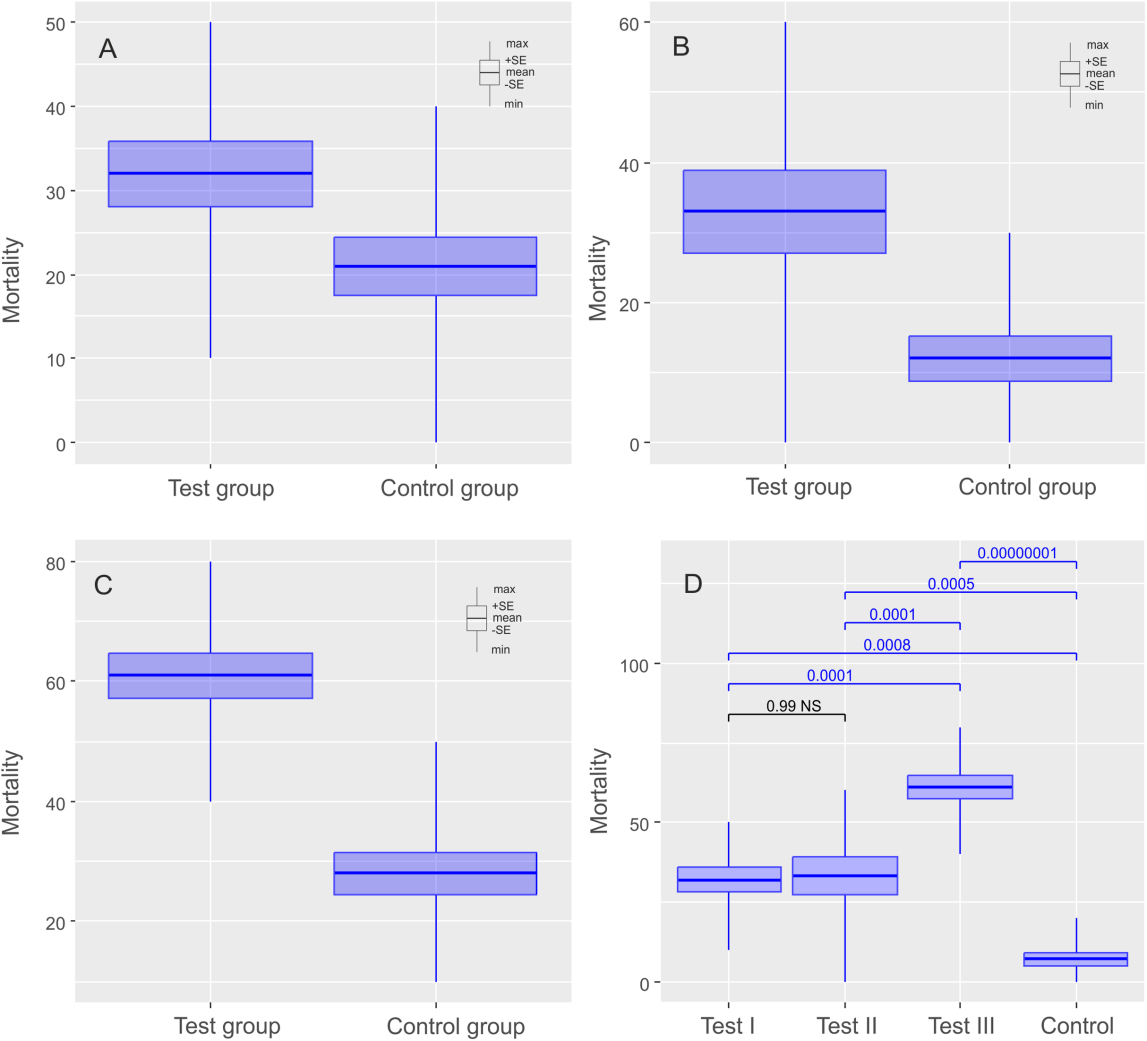
Results of experiments on *Milnesium inceptum* Morek et al., 2019, shown as comparisons of mortalities in test and control groups. The diagrams show (A) comparisons of mortalities of anhydrobiotic specimens, (B) comparisons of mortalities of the specimens during entering into cryptobiosis, (C) comparisons of mortalities of the specimens returning to an active state, and (D) the comparison of experiments. Every diagram shows the difference in mortality (expressed as the percentage of dead individuals) between the Test Group and the Control Group. Blue indicates statistically significant differences, while gray indicates statistically insignificant differences. Max, maximal value; Min, minimal value; Mean, mean value and SE, standard error.

The mean (±SE) percentage of mortality in experimental groups was 46.00% (±5.41%) and 32.00% (±3.88%) for *Ech. testudo* and *Mil. inceptum*, respectively. On the other hand, mortality in control groups was clearly lower and it amounted to 29.00% (±4.33%) for *Ech. testudo* (Table 1) and 21.00% (±3.48%) for *Mil. inceptum* (Table 1). The differences were statistically significant (*Ech. testudo*: *t*_17_ = 2.45, *p* = 0.025, Fig. 2A, and *Mil. inceptum*: *t*_17_ = 2.18, *p* = 0.045, Fig. 3A).

**Table 1:**
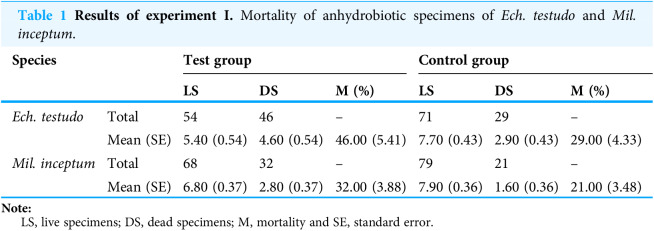
Results of experiment I. Mortality of anhydrobiotic specimens of *Ech. testudo* and *Mil. inceptum*.

Species		Test group			Control group		
		LS	DS	M (%)	LS	DS	M (%)
*Ech. testudo*	Total	54	46	–	71	29	–
	Mean (SE)	5.40 (0.54)	4.60 (0.54)	46.00 (5.41)	7.70 (0.43)	2.90 (0.43)	29.00 (4.33)
*Mil. inceptum*	Total	68	32	–	79	21	–
	Mean (SE)	6.80 (0.37)	2.80 (0.37)	32.00 (3.88)	7.90 (0.36)	1.60 (0.36)	21.00 (3.48)

**Note:**

LS, live specimens; DS, dead specimens; M, mortality and SE, standard error.

### Experiment II: testing the influence of hypomagnetic conditions on tardigrades in the process of entering anhydrobiosis

In this case, the mean (±SE) percentage of mortality in experimental groups was 38.00% (±6.11%) for *Ech. testudo* (Table 2) and 33.00% (±5.97 %) for *Mil. inceptum* (Table 2). Again, mortality in control groups was clearly lower 24.00% (±5.20%) for *Ech. testudo* (Table 2) and 12.00% (±3.26 %) for *Mil. inceptum* (Table 2). However, the differences were statistically significant only for *Mil. inceptum* (*t*_17_ = 3.08, *p* = 0.008, Fig. 2B), and not for *Ech. testudo* (*t*_17_ =1.74, *p* = 0.09, Fig. 3B).

**Table 2:** Results of experiment II. Mortality of the *Ech. testudo* and *Mil. inceptum* specimens entering anhydrobiosis.

Species		Test group			Control group		
		LS	DS	M (%)	LS	DS	M (%)
*Ech. testudo*	Total	62	38	–	76	24	–
	Mean (SE)	6.20 (0.61)	3.80 (0.61)	38.00 (6.11)	7.60 (0.52)	2.40 (0.52)	24.00 (5.20)
*Mil. inceptum*	Total	67	33	–	88	12	–
	Mean (SE)	6.70 (0.59)	3.30 (0.59)	33.00 (5.97)	8.8 (0.32)	1.20 (0.32)	12.00 (3.26)

**Note:**

LS, live specimens; DS, dead specimens; M, mortality and SE, standard error.

### Experiment III: testing the influence of hypomagnetic conditions on tardigrades returning from anhydrobiosis to the active state

The mean (±SD) percentage of mortality in experimental groups was 59.00% (±4.06%) for *Ech. testudo* (Table 3) and 61.00% (±3.78%) for *Mil. inceptum* (Table 3). Mortality in control groups was clearly lower, that is, 20.00% (±4.47%) for *Ech. testudo* (Table 3) and 28.00% (±3.59%) for *Mil. inceptum* (Table 3). The differences were statistically significant for both *Ech. testudo ( t*_17_ = 6.45, *p* = 0.0000001, Fig. 2C) and *Mil. Inceptum* (*t*_17_ = 6.32, *p* = 0.0000001, Fig. 3C).

**Table 3:**
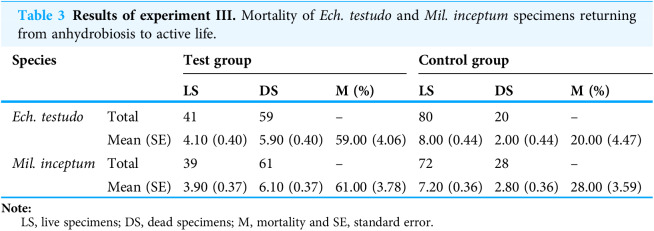
Results of experiment III. Mortality of *Ech. testudo* and *Mil. inceptum* specimens returning from anhydrobiosis to active life.

Species		Test group			Control group		
		LS	DS	M (%)	LS	DS	M (%)
*Ech. testudo*	Total	41	59	–	80	20	–
	Mean (SE)	4.10 (0.40)	5.90 (0.40)	59.00 (4.06)	8.00 (0.44)	2.00 (0.44)	20.00 (4.47)
*Mil. inceptum*	Total	39	61	–	72	28	–
	Mean (SE)	3.90 (0.37)	6.10 (0.37)	61.00 (3.78)	7.20 (0.36)	2.80 (0.36)	28.00 (3.59)

**Note:**

LS, live specimens; DS, dead specimens; M, mortality and SE, standard error.

### A comparison of survival rates depending on the moment of isolation from the geomagnetic field

We found differences between all three experiments for both species (*Ech. testudo*: one-way ANOVA *F*_2,27_ = 13.15, *p* = 0.00010, *Mil. inceptum* one-way ANOVA *F*_2.36_ = 27.96, *p* = 0.000000001), but there was no general pattern. According to the post-hoc Tukey test, the largest differences for *Ech. testudo* were observed between third experiment and the control group (post-hoc Tukey: *p* < 0.0000001), the second and the third experiment (post-hoc Tukey: *p* = 0.00001). Differences between the first experiment and the control group (post-hoc Tukey: *p* = 0.000005) and between the first and the second experiment (post-hoc Tukey: *p* = 0.006),were smaller, but still statistically significant (Fig. 2D; Data S4). On the other hand, differences for *Mil. inceptum* between particular experimental groups were statistically significant (in all cases post-hoc Tukey: *p* < 0.0001) as shown in Fig. 3D (Data S5) except the first experiment vs the second experiment as no significant differences were found (post-hoc Tukey: *p* = 0.99). Differences recorded between the first and the third experiment, the second and the third were equally high (post-hoc Tukey: *p* = 0.0001). The smallest difference was observed between the first experiment and the control group (post-hoc Tukey: *p* = 0.0008).

## Discussion

The results obtained in experiments on heterotardigrade *Ech. testudo* and eutardigrade *Mil. inceptum* clearly showed that even partial isolation from the geomagnetic field, that is, hypomagnetic conditions, has negative influence on anhydrobiotic abilities of tardigrades. The general trend remains similar to the results obtained for *Hys. exemplaris* (Erdmann et al., 2017), however, significant differences in the reaction to hypomagnetic conditions were observed among representatives of different tardigrade species.

In studies conducted on *Hys. exemplaris*, we identified two critical moments for anhydrobiosis abilities, that is, while entering anhydrobiosis, and upon returning to the active state. Both processes were strongly affected by hypomagnetic conditions. However, no influence of hypomagnetic conditions was observed for specimens in the state of anhydrobiosis (Erdmann et al., 2017). This observation partially contradicts the results for *Ech. testudo* and *Mil. inceptum*, because both species demonstrated a negative reaction to hypomagnetic conditions not only while entering anhydrobiosis and returning to the active state, but also in the state of anhydrobiosis.

It is well known that hypomagnetic conditions affect metabolic and biochemical processes of other organisms (Dubrov, 1978; Conley, 1970; Tombarkiewicz, 2008; Janicki, 2008; Baek et al., 2019). But, due to a current knowledge tardigrade metabolism during anhydrobiosis is significantly decreased or even stopped (Pigoń & Węglarska, 1953, 1955; Clegg, 1973; Hengherr, Brümmer & Schill, 2008; Schill & Hengherr, 2018), that is connected with “Sleeping Beauty” hypothesis which assumes that during cryptobiosis tardigrades do not age (for review see Kaczmarek et al., 2019). Therefore, no differences among anhydrobiotic tardigrades in normal and hypomagnetic conditions should occur. However, even if the “Sleeping Beauty” model of anhydrobiosis is correct, and most biochemical processes, including ageing, are stopped, tardigrades are still affected by hypomagnetic conditions, due to potential cellular damage caused by chemical ageing (Schill & Hengherr, 2018). Our results can suggest that although metabolism during anhydrobiosis is probably very low, it is still probably on the level which can be affected by hypomagnetic conditions.

Results of experiments II and III obtained for *Mil. inceptum* are very similar to the results conducted on *Hys. exemplaris* (Erdmann et al., 2017), whereas results obtained for *Ech. testudo* were contradictory. In the case of *Mil. inceptum*, both experiments demonstrated that the two critical moments for this species happened upon entering anhydrobiosis (experiment II) and returning to the active state (experiment III), because hypomagnetic conditions significantly increased mortality in the test groups. In the case of *Ech. testudo*, we noticed significant differences between the control and test group in experiment III, and the obtained differences were even higher than for *Mil. inceptum*. However, differences found between the test and the control group of *Ech. testudo* in experiment II were not statistically significant. It can mean that *Ech. testudo* is less resistant to hypomagnetic conditions while returning to the active state than *Mil. inceptum* or *Hys. exemplaris*, but more resistant to hypomagnetic conditions while entering anhydrobiosis. Mortality trends in response to hypomagnetic conditions are similar for both of the studied species, and additionally *Hys. exemplaris*, that is, the highest mortality was observed in response to hypomagnetic conditions, but the mean mortality of *Ech. testudo* and *Mil. inceptum* was clearly lower in comparison to *Hys. exemplaris* (Erdmann et al., 2017). These results are connected with generally higher anhydrobiotic abilities of *Ech. testudo* and *Mil. inceptum*, which are terrestrial species inhabiting xerothermic habitats, as opposed to *Hys. exemplaris* which is a freshwater species with a low anhydrobiotic ability (Wright, 1989).

All these results lead to a conclusion that hypomagnetic conditions increase tardigrade mortality rate during anhydrobiosis. However, there are certain differences among different species, especially those with varying anhydrobiotic abilities and inhabiting different microhabitats.

Our research demonstrates that the influence of hypomagnetic conditions, often neglected in studies focused on potential survivability in space vacuum (for review see for example, Guidetti et al., 2012; Erdmann & Kaczmarek, 2017), should also be considered in future research on extremophiles, including tardigrades, to test their abilities to survive in the outer space or on different planets or moons. It is of highest importance, because hypomagnetic conditions significantly increase mortality of all studied tardigrade species. What else is equally important is the choice of a possible model species for further astrobiological studies. This is well visible in the case of *Hys. exemplaris* which is often used as a model species in different types of research (Gabriel et al., 2007; Bemm et al., 2016; Fernandez et al., 2016; Hering et al., 2016; Hyra et al., 2016; Kosztyła et al., 2016). Its low anhydrobiotic abilities and low tolerance to hypomagnetic conditions indicate that it should be replaced by other species, as results obtained in studies on *Hys. exemplaris* fail completely to be representative for Tardigrada.

Tardigrades are used increasingly in space research, mainly because of their high resistance to many environmental stressors (see for example, Guidetti et al., 2012; Erdmann & Kaczmarek, 2017). In the past not only astrobiologists, but also space law experts considered the risk of contaminating other celestial bodies with terrestrial life as the consequence of space exploration (Committee on Space Research (COSPAR), 2002; National Research Council, 2006).

This risk assessed in purely theoretical considerations became not so far-fetched when in 2019 an Israeli spacecraft, Beresheet, crashed on the Moon with tardigrades onboard (NASA Space Science Data Coordinated Archive, 2020; Møbjerg et al., 2020; Shahar & Greenbaum, 2020). What is more, according to COSPAR guidelines, Moon missions fall under category II, and requirements for preventing biological contamination are low and no sterilisation of the entire lander, using of clean rooms, or even inventory of organic samples is needed. What is particularly disturbing is the fact that SpaceIL, an Israeli organisation established in 2011 competing in the Google Lunar X Prize (GLXP) contest to land a spacecraft on the Moon, was unaware that tardigrades along with other biological samples had been added to a payload by the US-based Arch Mission Foundation. Nevertheless, the risk of contaminating the Moon by tardigrades was very low, because tardigrades were closed in a hermetic multi-layered metal box. Even if the container had been damaged during the collision, it would have been very unlikely for the tardigrades in the cryptobiotic state to return to activity and survive in such adverse environmental conditions, especially the lack of liquid water (Caplin, 2019). Although we do not know what happened with the tardigrades and the chances of their survival seemed small, the Beresheet spacecraft incident has sparked a new discussion on this topic (Caplin, 2019; Vidaurri, 2019; Shahar & Greenbaum, 2020), reminding us on exercising caution in space exploration. Currently, we know that at least several tardigrade species are able to survive conditions of space vacuum and possible conditions on some celestial bodies in the Solar System (for review see Guidetti et al., 2012; Erdmann & Kaczmarek, 2017; Jagadeesh, Roszkowska & Kaczmarek, 2018). Therefore, a question arises if it is possible that the influence of hypomagnetic conditions could be sufficient to completely eradicate the threat of space contamination by terrestrial extremophilic lifeforms. The response is ‘probably not’, but such a possibility might not be entirely excluded. The reason for such a reservation is that so far there have been limited studies on the influence of hypomagnetic conditions on tardigrade survivability, and none on other extremophilic invertebrates, such as rotifers, crustaceans or nematodes. The only papers on the influence of the magnetic field on nematodes concerns their ability to magneto-detection and magneto-navigation (Vidal-Gadea et al., 2015). Most studies on the influence of hypomagnetic conditions on terrestrial organisms, especially in the context of spaceflights, were conducted on vertebrates, that are, mice, rats and humans, or their isolated tissues (Conley, 1970; Binhi & Sarimov, 2009; Xiao et al., 2009; Fesenko et al., 2010; Binhi, 2012; Gurfinkel et al., 2016), and there were a few studies on crops/plants (Mo et al., 2011). The only organisms discovered to have higher tolerance to some environmental stressors were Bacteria or Archaea. However, even then researchers focused mostly on model organisms, such as *Escherichia coli* Escherich, 1885, and some pathogenic strains/species of *Shigella sonnei* (Levine, 1920; Weldin, 1927, *Salmonella* sp. or *Staphylococcus* sp. (Dubrov, 1978), *Deinococcus radiodurans* Brooks & Murray, 1981) or *Leptolyngbya laminose* (Gomont) (Anagnostidis & Komárek, 1988 ; Mastrapa et al., 2001; De La Vega, Rettberg & Reitz, 2007, De Vera et al., 2013). The scarcity of the above studies makes results of our experiments even more important, because they show that despite higher mortality of tardigrade species in hypomagnetic conditions, their chances to survive are not reduced to zero. It should be remembered that many species of tardigrades including *Hys. exemplaris, Ech. testudo* and *Mil. inceptum* are parthenogenetic (Nelson, Guidetti & Rebecchi, 2015), which means that even one specimen that survives a voyage in hypomagnetic conditions and finds favourable conditions can still establish a new population able to function in a normal way.

## Conclusion

In conclusion, although the current knowledge on the influence of hypomagnetic conditions on mortality of cryptobiotic organisms is still limited to tardigrades, our results suggest that the presence of the magnetic field is a very important factor should be considered in further research both in the context of potential space contamination and terrestrial organisms surviving on other planets and moons.

## Supplemental Information

10.7717/peerj.10630/supp-1 Supplemental Information 1 Temperature data for experiments I–III carried out on both *Ech. testudo and Mil. inceptum*. Temperature data used to calculate the average temperature inside the CIMF chamber. The table contains temperature data for 10:00 am and 10:00 pm, each day in every 24-day period (3 days of desiccation time period and 21 days of experimental time), for experiments I–III carried out on both*Ech. testudo and Mil. inceptum*.

10.7717/peerj.10630/supp-2 Supplemental Information 2 Summary of raw data from experiments I–III carried out on tardigrades of the species *Ech. testudo*. The data includes the number of dead individuals, the number of alive individuals and the percentage mortality of the tested replicates.

10.7717/peerj.10630/supp-3 Supplemental Information 3 Summary of raw data from experiments I–III carried out on tardigrades of the species *Mil. inceptum*.. The data includes the number of dead individuals, the number of alive individuals and the percentage mortality of the tested replicates.

10.7717/peerj.10630/supp-4 Supplemental Information 4 Comparison of experiments I–III carried out on tardigrades of the species *Ech. testudo*. The data includes comparisons between all three experiments I–III and control. The control group in this case, was expressed as the minimum values for each dish from the control groups for experiment. diff, differences between groups; lwr, lower 95% confidence level; upr, upper 95% confidence level; p adj, *p* value.

10.7717/peerj.10630/supp-5 Supplemental Information 5 Comparison of experiments I–III carried out on tardigrades of the species *Mil. inceptum*. The data includes comparisons between all three experiments I–III and control. The control group in this case, was expressed as the minimum values for each dish from the control groups for experiment. diff, differences between groups; lwr, lower 95% confidence level; upr, upper 95% confidence level; p adj, *p* value.

## Additional Information and Declarations

### Data Availability

The following information was supplied regarding data availability: The raw data are available in Supplemental Files S1, S2, and S3. The raw data in Supplemental File S1 show temperature data from the chamber. These data were used for determining the average temperature inside the chamber. The raw data in Supplemental Files S2 and S3 show the number of dead and alive specimens in test and control groups of all replications, of each experiment. These data were used for statistical analysis to compare differences in mortality between test and control groups.

## References

[ref-1] Altiero T, Guidetti R, Caselli V, Cesari M, Rebecchi L (2011). Ultraviolet-B radiation tolerance in hydrated and desiccated eutardigrades. Journal of Zoological Systematics and Evolutionary Research.

[ref-2] Anagnostidis K, Komárek J (1988). Modern approach to the classification system of cyanophytes: 3—Oscillatoriales. Archiv Fur Hydrobiologie.

[ref-3] Baek S, Choi H, Park H, Choo B, Kim S, Kim J (2019). Effects of a hypomagnetic field on DNA methylation during the differentiation of embryonic stem cells. Scientific Reports.

[ref-4] Bemm F, Weiß CL, Schultz J, Förster F (2016). Genome of a tardigrade: horizontal gene transfer or bacterial contamination?. Proceedings of the National Academy of Sciences of the United States of America.

[ref-5] Binhi VN (2012). Two types of magnetic biological effects: individual and batch effects. Biophysics.

[ref-6] Binhi  VN, Sarimov RM (2009). Zero magnetic field effect observed in human cognitive processes. Electromagnetic Biology and Medicine.

[ref-7] Binhi VN, Sarimov RM (2013). Effect of the hypomagnetic field on the size of the eye pupil. arXiv preprint arXiv:1302.2741.

[ref-8] Boothby TC (2018). Desiccation of *Hypsibius exemplaris*. Cold Spring Harbor Protocols.

[ref-9] Boothby TC, Tapia H, Brozena AH, Piszkiewicz S, Smith AE, Giovannini I, Rebecchi L, Pielak GJ, Koshland D, Goldstein B (2017). Tardigrades use intrinsically disordered proteins to survive desiccation. Molecular Cell.

[ref-10] Brooks BW, Murray RGE (1981). Nomenclature for *Micrococcus radiodurans* and other radiation-resistant cocci: *Deinococcaceae fam. nov*. and *Deinococcus gen. nov*., including five species. International Journal of Systematic Bacteriology.

[ref-11] Brothers JR, Lohmann KJ (2015). Evidence for geomagnetic imprinting and magnetic navigation in the natal homing of sea turtles. Current Biology.

[ref-12] Bryce DL (1892). On some moss-dwelling Cathypnadae; with descriptions of five new species. London: Science-Gossip.

[ref-13] Caplin N (2019). Anything to declare?. Physics World.

[ref-14] Chuss DT (2008). Cosmic background explorer, NASA goddard space flight center. https://lambda.gsfc.nasa.gov/product/cobe/.

[ref-15] Clegg JS, Crowe JH, Clegg JS (1973). Do dried cryptobiotes have a metabolism?. Anhydrobiosis.

[ref-16] Conley CC (1970). A review of the biological effects of very low magnetic fields—NASA technical note. http://ntrs.nasa.gov/archive/nasa/casi.ntrs.nasa.gov/19700024915.pdf.

[ref-17] Copley J (1999). Indestructible. New Scientist.

[ref-18] Committee on Space Research (COSPAR) (2002). COSPAR Planetary Protection Policy. https://web.archive.org/web/20130306111646/.

[ref-19] De La Vega UP, Rettberg P, Reitz G (2007). Simulation of the environmental climate conditions on Martian surface and its effect on *Deinococcus radiodurans*. Young Neutron Stars and Supernova Remnants.

[ref-20] De Vera JP, Dulai S, Kereszturi A, Koncz L, Pocs T (2013). Results on the survival of cryptobiotic cyanobacteria samples after exposure to Mars-like environmental conditions. International Journal of Astrobiology.

[ref-21] Ding HM, Wang X, Mo WC, Qin LL, Wong S, Fu JP, Tan Y, Liu Y, He RQ, Hua Q (2018). Hypomagnetic fields cause anxiety in adult male mice. Bioelectromagnetics.

[ref-22] Doyère M (1840). Memoire sur les tardigrades. *Annales des Sciences Naturelles*. Zoologie Series.

[ref-23] Dubrov AP (1978). The geomagnetic field and life—geomagnetobiology.

[ref-24] Erdmann W, Idzikowski B, Kowalski W, Szymański B, Kaczmarek Ł, Kosicki JZ, Kosicka E (2018). *Changes in expression level of transcripts encoding stress proteins in Tardigrades in hipomagnetic conditions*.

[ref-25] Erdmann W, Idzikowski B, Kowalski W, Szymański B, Kosicki JZ, Kaczmarek Ł (2017). Can the tardigrade *Hypsibius dujardini* survive in the absence of the geomagnetic field?. PLOS ONE.

[ref-26] Erdmann W, Kaczmarek Ł (2017). Tardigrades in space research—past and future. Origins Life and Evolution Biospheres.

[ref-27] Escherich T (1885). Die darmbakterien des neugeborenen und sauglings. Fortschritte der Medizin.

[ref-28] Fernandez C, Vasanthan T, Kissoon N, Karam G, Duquette N, Seymour C, Stone JR (2016). Radiation tolerance and bystander effects in the eutardigrade species *Hypsibius dujardini* (Parachaela: Hypsibiidae). Zoological Journal of the Linnean Society.

[ref-29] Fesenko EE, Mezhevikina LM, Osipenko MA, Gordon RY, Khutzian SS (2010). Effect of the “zero” magnetic field on early embryogenesis in mice. Electromagnetic Biology and Medicine.

[ref-30] Gabriel WN, McNuff R, Patel SK, Gregory TR, Jeck WR, Joes CD, Goldstein B (2007). The tardigrade *Hypsibius dujardini*, a new model for studying the evolution of development. Developmental Biology.

[ref-31] Gmünder FK, Suter RN, Kiess M, Urfer R, Nordau CG, Cogoli A (1989). Mammalian cell cultivation in space. Advances in Space Research.

[ref-32] Grigoriev AI, Potapov AN (2014). Approaches to the development of biomedical support systems for piloted exploration missions. Acta Astronautica.

[ref-33] Guidetti R, Rizzo AM, Altiero T, Rebecchi L (2012). What can we learn from the toughest animals of the Earth? Water bears (tardigrades) as multicellular model organisms in order to perform scientific preparations for lunar exploration. Planetary and Space Science.

[ref-34] Gurfinkel YI, Vasin AL, Matveeva TA, Sasonko ML (2016). Evaluation of the hypomagnetic environment effects on capillary blood circulation, blood pressure and heart rate. Human Physiology.

[ref-35] Gąsiorek P, Stec D, Morek W, Michalczyk Ł (2018). An integrative redescription of *Hypsibius dujardini* (Doyère, 1840), the nominal taxon for Hypsibioidea (Tardigrada : Eutardigrada). Zootaxa.

[ref-36] Gąsiorek P, Vončina K, Michalczyk Ł (2019). *Echiniscus testudo* (Doyère, 1840) in New Zealand: anthropogenic dispersal or evidence for the ‘Everything is Everywhere’ hypothesis?. New Zealand Journal of Zoology.

[ref-37] Hargreaves JK, Hargreaves JK (1995). The solar wind and the magnetosphere. The Solar-Terrestrial Environment: An Introduction to Geospace—the Science of the Terrestrial Upper Atmosphere, Ionosphere, and Magnetosphere.

[ref-38] Hashimoto T, Horikawa DD, Saito Y, Kuwahara H, Kozuka-Hata H, Shin-I T, Minakuchi Y, Ohishi K, Motoyama A, Aizu T, Enomoto A, Kondo K, Tanaka S, Hara Y, Koshikawa S, Sagara H, Miura T, Yokobori S, Miyagawa K, Suzuki Y, Kubo T, Oyama M, Kohara Y, Fujiyama A, Arakawa K, Katayama T, Toyoda A, Kunieda T (2016). Extremotolerant tardigrade genome and improved radiotolerance of human cultured cells by tardigrade-unique protein. Nature Communications.

[ref-39] Hengherr S, Brümmer F, Schill RO (2008). Anhydrobiosis in tardigrades and its effects on longevity traits. Journal of Zoology.

[ref-40] Hering L, Bouameur JE, Reichelt J, Magin TM, Mayer G (2016). Novel origin of lamin-derived cytoplasmic intermediate filaments in tardigrades. eLife.

[ref-41] Hyra M, Poprawa I, Włodarczyk A, Student S, Sosnakowska L, Kszuk-Jendrysik M, Rost-Roszkowska MM (2016). Ultrastructural changes in the midgut epithelium of *Hypsibius dujardini* (Doyère, 1840) (Tardigrada, Eutardigrada, Hypsibiidae) in relation to oogenesis. Zoological Journal of the Linnean Society.

[ref-42] Idzikowski B, Szajek A, Greneche JM, Kovac J (2004). Nanogranular Fe_x_Ni_23-x_B_6_ phase formation during devitrification of nickel-rich Ni_64_Fe_16_Zr_7_B_12_Au_1_ amorphous alloy. Applied Physics Letters.

[ref-43] Jagadeesh MK, Roszkowska M, Kaczmarek Ł (2018). Tardigrade indexing approach on exoplanets. Life Sciences in Space Research.

[ref-44] Janicki JS, Janicki JS (2008). Magnetobiologia, podstawowe procesy zachodzące w organizmie pod wpływem pola magnetycznego. Zastosowanie stałych pól magnetycznych w terapii.

[ref-45] Jönsson KI, Borsari S, Rebecchi L (2001). Anhydrobiotic survival in populations of the tardigrades *Richtersius coronifer* and *Ramazzottius oberhaeuseri* from Italy and Sweden. Zoologischer Anzeiger.

[ref-46] Jönsson KI, Rabbow E, Schill RO, Harms-Ringdahl M, Rettberg P (2008). Tardigrades survive exposure to space in low Earth orbit. Current Biology.

[ref-47] Jørgensen A, Faurby S, Persson DK, Halberg KA, Kristensen RM, Møbjerg N (2013). Genetic diversity in the parthenogenetic reproducing tardigrade *Echiniscus testudo* (Heterotardigrada: Echiniscoidea). Journal of Limnology.

[ref-48] Jørgensen A, Møbjerg N, Kristensen RM (2007). A molecular study of the tardigrade *Echiniscus testudo* (Echiniscidae) reveals low DNA sequence diversity over a large geographical area. Journal of Limnology.

[ref-49] Kaczmarek Ł, Michalczyk Ł, McInnes SJ (2015). Annotated zoogeography of non-marine Tardigrada—part II: South America. Zootaxa.

[ref-50] Kaczmarek Ł, Michalczyk Ł, McInnes SJ (2016). Annotated zoogeography of non-marine Tardigrada—part III: North America and Greenland. Zootaxa.

[ref-51] Kaczmarek Ł, Roszkowska M, Fontaneto D, Jezierska M, Pietrzak B, Wieczorek R, Poprawa I, Kosicki JZ, Karachitos A, Kmita H (2019). Staying young and fit? Ontogenetic and phylogenetic consequences of animal anhydrobiosis. Journal of Zoology.

[ref-52] Kondo K, Kubo T, Kunieda T (2015). Suggested involvement of PP1/PP2A activity and de novo gene expression in anhydrobiotic survival in a tardigrade, *Hypsibius dujardini*, by chemical genetic approach. PLOS ONE.

[ref-53] Kopcewicz M, Grabias A, Škorvánek I, Marcin J, Idzikowski B (1999). Mössbauer study of the magnetic properties of nanocrystalline Fe_80.5_Nb_7_B_12.5_ alloy. Journal of Applied Physics.

[ref-54] Kosztyła P, Stec D, Morek W, Gąsiorek P, Zawierucha K, Michno K, Ufir K, Małek D, Hlebowicz K, Laska A, Dudziak M, Frohme M, Prokop ZM, Kaczmarek Ł, Michalczyk Ł (2016). Experimental taxonomy confirms the environmental stability of morphometric traits in a taxonomically challenging group of microinvertebrates. Zoological Journal of the Linnean Society.

[ref-55] Langbein D (1986). Physical parameters affecting living cells in space. Advances in Space Research.

[ref-56] Levine M (1920). Dysentery and allied bacilli. Journal of Infectious Diseases.

[ref-57] Mastrapa RME, Glanzberg H, Head JN, Melosh HJ, Nicholson WL (2001). Survival of bacteria exposed to extreme acceleration: Implications for panspermia. Earth and Planetary Science Letters.

[ref-58] Maupas É (1900). Modes et formes de reproduction des nematodes. Archives de Zoologie Expérimentale et Générale.

[ref-59] McInnes SJ (1994). Zoogeographic distribution of terrestrial/freshwater tardigrades from current literature. Journal of Natural History.

[ref-60] McInnes SJ, Michalczyk Ł, Kaczmarek Ł (2017). Annotated zoogeography of non-marine Tardigrada—part IV: Africa. Zootaxa.

[ref-61] Miglierini M, Tóth I, Seberíni M, Illeková E, Idzikowski B (2002). Structure and hyperfine interactions of melt-spun Fe_80_Mo_7_X_1_B_12_ (X = Cu or Au) before and after transformation into nanocrystalline states. Journal of Physics: Condensed Matter.

[ref-62] Mo WC, Liu Y, Bartlett PF, He RQ (2014). Transcriptome profile of human neuroblastoma cells in the hypomagnetic field. Science China-life Sciences.

[ref-63] Mo WC, Liu Y, He RQ (2014). Hypomagnetic field, an ignorable environmental factor in space?. Science China Life Sciences.

[ref-64] Mo WC, Zhang ZJ, Liu Y, Zhai GJ, Jiang YD, He RQ (2011). Effects of a hypogeomagnetic field on gravitropism and germination in soybean. Advances in Space Research.

[ref-65] Morek W, Suzuki AC, Schill RO, Georgiev D, Yankova M, Marley NJ, Michalczyk Ł (2019). Redescription of *Milnesium alpigenum* Ehrenberg, 1853 (Tardigrada: Apochela) and a description of *Milnesium inceptum* sp. nov., a tardigrade laboratory model. Zootaxa.

[ref-66] Morrow RC, Bula RJ, Tibbitts TW, Dinauer WR (1994). The astroculture flight experiment series, validating technologies for growing plants in space. Advances in Space Research.

[ref-67] Møbjerg N, Michalczyk Ł, Mcinnes SJ, Christenhusz MJM (2020). Research presented at the 14th International Symposium on Tardigrada: progress in studies on water bears. Zoological Journal of the Linnean Society.

[ref-68] NASA Space Science Data Coordinated Archive (2020). Beresheet mission, NSSDCA/COSPAR ID: 2019–009B. https://nssdc.gsfc.nasa.gov/nmc/spacecraft/display.action?id=2019-009B.

[ref-69] National Research Council (2006). Preventing the forward contamination of Mars.

[ref-70] Nelson DR, Guidetti R, Rebecchi L, Thorp JH, Rogers DC (2015). Phylum Tardigrada (chapter 17). Thorp and Covich’s Freshwater Invertebrates, 4th edn—Ecology and General Biology.

[ref-71] Osipenko MA, Mezhevikina LM, Krasts IV, Iashin VA, Novikov VV, Fesenko EE (2008). Influence of “zero“ magnetic field on the growth of embryonic cells and primary embryos of mouse in vitro. Biofizika.

[ref-72] Persson D, Halberg KA, Jørgensen A, Ricci C, Møbjerg N, Kristensen RM (2011). Extreme stress tolerance in tardigrades: surviving space conditions in low earth orbit. Journal of Zoological Systematics and Evolutionary Research.

[ref-73] Pigoń A, Węglarska B (1953). The respiration of Tardigrada: a study in animal anabiosis. Bulletin L’Académie Polonaise des Science.

[ref-74] Pigoń A, Węglarska B (1955). Rate of metabolism in tardigrades during active life and anabiosis. Nature.

[ref-75] Ramlov H, Westh P (2001). Cryptobiosis in the eutardigrade *Adorybiotus (Richtersius) cornifer*: Tolerance to alcohols, temperature and de novo protein synthesis. Zoologischer Anzeiger—A Journal of Comparative Zoology.

[ref-76] Rebecchi L, Altiero T, Cesari M, Marchioro T, Giovannini I, Rizzo AM, Ganga PL, Vikich M, Donati A, Zolesi V, Bertolani R, Guidetti R (2011a). TARDIKISS: tardigrades in the mission STS-134, the last of the shuttle Endeavour.

[ref-77] Rebecchi L, Altiero T, Guidetti R, Caselli V, Cesari M (2011b). Resistance of the anhydrobiotic eutardigrade *Paramacrobiotus richtersi* to space flight (LIFE– TARSE mission on FOTON-M3). Journal of Zoological Systematics and Evolutionary Research.

[ref-78] Rebecchi L, Altiero T, Guidetti R, Cesari M, Bertolani R, Negroni M, Rizzo AM (2009a). Tardigrade resistance to space effects: first results of experiments on the LIFE-TARSE mission on FOTON-M3. Astrobiology.

[ref-79] Rebecchi L, Cesari M, Altiero T, Frigieri A, Guidetti R (2009b). Survival and DNA degradation in anhydrobiotic tardigrades. Journal of Experimental Biology.

[ref-80] Rebecchi L, Guidetti R, Borsari S, Altiero T, Bertolani R (2006). Dynamics of long-term anhydrobiotic survival of lichen-dwelling tardigrades. Hydrobiologia.

[ref-81] Roszkowska M, Gołdyn B, Grobys D, Kmita H, Kosicki JZ, Stec D, Kaczmarek Ł (2018). Differences in tolerance to anhydrobiotic conditions among tardigrade species.

[ref-82] Sarimov RM, Binhi VN, Milyaev VA (2008). The influence of geomagnetic field compensation on human cognitive processes. Biophysics.

[ref-83] Schill RO, Hengherr S, Schill RO (2018). Environmental adaptations: desiccation tolerance. Water Bears: the Biology of Tardigrades.

[ref-84] Shahar K, Greenbaum D (2020). Lessons in space regulations from the lunar tardigrades of the Beresheet hard landing. Nature Astronomy.

[ref-85] Sibeck DG, Lin RQ (2014). Size and shape of the distant magnetotail. Journal of Geophysical Research: Space Physics.

[ref-86] Stec D, Smolak R, Kaczmarek Ł, Michalczyk Ł (2015). An integrative description of *Macrobiotus paulinae* sp. nov. (Tardigrada: Eutardigrada: Macrobiotidae: *hufelandi* group) from Kenya. Zootaxa.

[ref-87] Tombarkiewicz B (2008). Effect of long-term geomagnetic field deprivation on the concentration of some elements in the hair of laboratory rats. Environmental Toxicology and Pharmacology.

[ref-88] Vidal-Gadea A, Ward K, Beron C, Ghorashian N, Gokce S, Russell J, Truong N, Parikh A, Gadea O, Ben-Yakar A, Pierce-Shimomura J (2015). Magnetosensitive neurons mediate geomagnetic orientation in *Caenorhabditis elegans*. eLife.

[ref-89] Vidaurri M (2019). Building an ethical consensus. Physics World.

[ref-90] Vukich M, Ganga PL, Cavalieri D, Rivero D, Pollastri S, Mugnai S, Mancuso S, Pastorelli S, Lambreva M, Antonacci A, Margonelli A, Bertalan I, Johan- Ningmeier U, Giardi MT, Rea G, Pugliese M, Quarto M, Roca V, Zanin A, Borla O, Rebecchi L, Altiero T, Guidetti R, Cesari M, Marchioro T, Bertolani R, Pace E, De Sio A, Casarosa M, Tozzetti L, Branciamore S, Gallori E, Scarigella M, Bruzzi M, Bucciolini M, Talamonti C, Donati A, Zolesi V (2012). BIOKIS: a model payload for multisciplinary experiments in microgravity. Microgravity Science and Technology.

[ref-91] Wang XB, Xu ML, Li B, Li DF, Jiang JC (2003). The taste of oneday- old chicks incubated in hypomagnetic field avoids long-term memory impairment. Science Bulletin.

[ref-92] Wei-Chuan MO, Jing-Peng FU, Hai-Min DING, Qian HUA, Rong-Qiao HE (2015). Hypomagnetic field alters circadian rhythm and increases algesia in adult male mice. PIBB.

[ref-93] Weldin JC (1927). The colon-typhoid group of bacteria and related forms—relationships and classification. Iowa State Journal of Science.

[ref-94] Wiltschko W, Wiltschko R (2005). Magnetic orientation and magnetoreception in birds and other animals. Journal of Comparative Physiology A.

[ref-95] Wright JC (1989). Desiccation tolerance and water retentive mechanisms in tardigrades. Journal of Experimental Biology.

[ref-96] Xiao Y, Wang Q, Xu ML, Jiang JC, Li B (2009). Chicks incubated in hypomagnetic field need more exogenous noradrenaline for memory consolidation. Advances in Space Research.

[ref-97] Yosef R, Raz M, Ben-Baruch N, Shmueli L, Kosicki JZ, Fratczak M, Tryjanowski P (2020). Directional preferences of dogs’ changes in the presence of a bar magnet: educational experiments in Israel. Journal of Veterinary Behavior.

[ref-98] Zaporozhan VM, Nasibullin BA, Gozhenko AI, Shapranov RA (2002). Effects of hypogeomagnetic fields on the structural functional activity of rat cerebral cortex. Fiziologichnyi Zhurnal.

[ref-99] Zar JH (1999). Biostatistical analysis.

[ref-100] Zhang B, Lu H, Xi W, Zhou X, Xu S, Zhang K, Jiang J, Li Y, Guo A (2004). Exposure to hypomagnetic field space for multiple generations causes amnesia in *Drosophila melanogaster*. Neuroscience Letters.

